# Forest frontiers out of control: The long-term effects of discourses, policies, and markets on conservation and development of the Brazilian Amazon

**DOI:** 10.1007/s13280-021-01637-4

**Published:** 2021-10-12

**Authors:** Benno Pokorny, Pablo Pacheco, Wil de Jong, Steffen Karl Entenmann

**Affiliations:** 1 Faculty of Environment and Natural Resources, University of Freiburg (Germany), Tennenbacher Strasse 4, 79106 Freiburg, Germany; 2 World Wide Fund for Nature, 1250 24th St NW, Washington, DC 20037 USA; 3 Center for Southeast Asian and Integrated Area Studies, Kyoto University (Japan), 46 Shimoadachichou, Sakyoku, Kyoto, 606-8501 Japan; 4 Chair of Silvicutlure, Faculty of Environment and Natural Resources, University of Freiburg (Germany), Tennenbacher Strasse 4, 79106 Freiburg, Germany

**Keywords:** Agricultural frontiers, Amazon region, Deforestation, Discourses, Policies, Rural development

## Abstract

With the Brazilian military governments of the 1960s, systematic economic development of the Amazon began. Social and environmental concerns have entered Amazonian discourses and policies only since the 1990s. Since then, reports of threats to forests and indigenous people have alternated with reports of socio-economic progress and environmental achievements. These contradictions often arise from limited thematic, sectoral, temporal, or spatial perspectives, and lead to misinterpretation. Our paper offers a comprehensive picture of discourses, policies, and socio-environmental dynamics for the entire region over the last five decades. We distinguish eight historical policy phases, each of which had little effect on near-linear dynamics of demographic growth and land-use expansion, although some policies showed the potential to change the course of development. To prevent local, national, and international actors from continuing to assert harmful interests in the region, a coherent long-term commitment and change in the collective mindset are needed.

## Introduction

The use of land and the extraction of natural resources by human societies has in many locations and at many moments in history brought with it concerns about our planet’s finite capacity to sustain our survival, development, economic growth, and social reproduction (Steffen et al. 2015). Ecological thinking was a cultural attribute among people living in close vicinity of and dependent on nature, but not something that influenced national political decision-making until only recently. The works of pioneering thinkers such as William Vogt (1948) and Rachel Carson (1962) affected only a small number of experts and triggered little political response. This changed in the late 1960s with the first space pictures of our *blue planet*. In 1972, Meadow's seminal book *Limits of Growth* had an enormous echo, and the *oil shock* in 1973 aggravated societal concern about the finite character of natural resources. In the face of looming climate change, environmental conservation discourses have proliferated in mainstream political debates, which impressively became evident at the United Nations Conference on Environment and Development (UNCED), in Rio de Janeiro in 1992. Pressured by emerging grassroots movements, the international community also recognized that human rights and environmental justice needed to be part of the sustainable development paradigm to assure that rights and well-being of indigenous and traditional forest residents are respected (UN 2007).

**Fig. 1:**
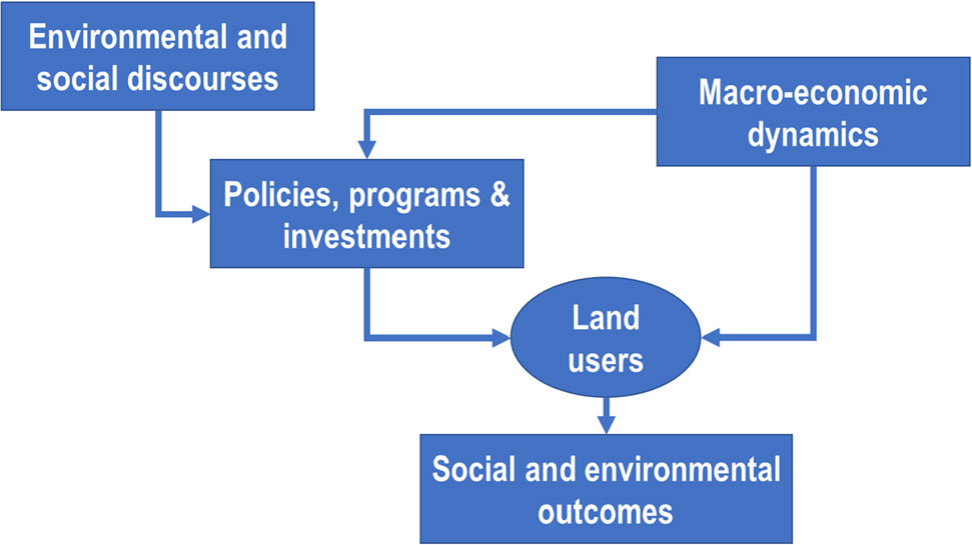
Conceptual understanding of the impact pathway from discourses to territorial outcomes

Since UNCED, discourses of sustainable development reconciling the goals of environmental justice, environmental protection, and economic growth have competed for prominence (Arts and Buizer 2009; Arts et al. 2010; UNDP 2013). Discourses, as understood here, refer to confined ideas that emerge and are shared among specialists in a certain field, or members of groups who share a certain interest. Discourses are stated and restated by adherents in public speeches and media (Arts and Buizer 2009) and are produced and reproduced for a national audience (e.g., Medina et al. 2009) or an international public (Arts et al. 2010). To what extent social and environmental discourses have influenced policy and economic action at global, national, and local levels is debated. Some scholars argue that environmental discourses have been translated into investments in forest conservation and the enactment of related environmental policies and institutions (Kleinschmit et al. 2009; Arts et al. 2010; Abranches 2014), which, over time, led to policy and land-use decisions that reduced deforestation (Nepstad et al. 2006a, b, 2014; Schwartzmann et al. 2010; Assunção et al. 2015) (Fig. 1).

Others argue that environmental conservation agendas are overshadowed by national economic growth priorities often shaped by large public investment programs (Hecht 2005; Crespo Cuaresma et al. 2017). Private investments intend to meet profit expectations by entrepreneurs and shareholders (Arestis and Saad-Filho 2007; Kröger 2020), consumption needs of affluent consumers in large parts of the globe (Fróna et al. 2019), and livelihood demands of natural resource users (van Vliet et al. 2013; Jakovac et al. 2017). The literature also suggests that macroeconomic conditions such as commodity prices and exchange rates have a major influence on land-based investments since investors and farmers make decisions based on profit expectations (Ellis, 2003; Williamson 2004). Another common argument is that particularly economic stability favors long-term investments in economically competitive sectors, including agriculture (Yanıkkaya and Taner 2019), and, in contrast, times of crises hamper economic investments, which may reduce pressures on ecosystems, but negatively affects well-being (Ötker and Podpiera 2013). In addition, it is hypothesized that the periods of economic growth accompanies accelerated environmental destruction (Crespo Cuaresma et al. 2017).

Nowhere else is this debate livelier than in the Brazilian Amazon. Following the UNCED, policy makers and environmental experts expected that concerted efforts of governments, civil society, and the private sector aided by the international cooperation for the conservation of Amazonian forests would result in a significant decrease in deforestation and progress in terms of environmental justice (Carvalho et al. 2019). Indeed, some studies report impressive achievements of reduced deforestation (Brannstrom 2009; Nepstad et al. 2014; Börner and West 2018; Simonet et al. 2018), recognized tenure rights to indigenous people and extractive communities (Blackman and Veit 2018), progress in the demarcation of protected areas (Pfaff et al. 2015), and the promotion of environmentally sound land use (FAO 2014). Brazil has even been referred as an example of the harmonization of economic growth-oriented neoliberal policies with the conservation of forests (Scholz and Schönenberg 2007; Macedo et al. 2012; Stabile et al. 2020)—yet these views have been questioned due to a worsening of environmental performance and threats to indigenous rights during the recent past. Others, however, point out that economic growth policies and business models that externalize negative environmental impacts continue pushing agricultural frontiers forward with devastating effects on forests and forest-dependent populations (World Bank Group 2016; Toledo et al. 2017), which include ethnic indigenous groups as well as traditional communities of *caboclos* and *riberenhos* (Barretto Filho 2006).

Against this backdrop, the paper aims to better understand whether and to what degree, within the wider regional macroeconomic dynamics, the emerging environmental conservation, and environmental justice discourses, conservation policies, programs, and public and private investments in the Brazilian Amazon have influenced environmental and social outcomes. It asks if—and to what degree—these discourses, policies, and investments have succeeded in influencing the economic actors active in the region to adapt their land-use decisions to support desired social and environmental outcomes. To find evidence for and against the existence of such causal linkages, we reviewed the discourses, policies, programs, investments, and macroeconomic dynamics relevant to the region over the last 50 years.

## Methods

To find evidence for the hypothesis that the proliferating environmental conservation and justice discourses have influenced regional development policies, and therefore yielded positive socio-environmental effects, we undertook a historical analysis of Brazil’s national macroeconomic and the Amazon’s regional dynamics since the 1970s, since when the Brazilian Amazon began to capture federal policymakers’ attention. By considering a 50-year time span, we aim to avoid typical biases of studies with narrow thematic, spatial, or temporal focuses (Popper 2002). For the same reason, our analysis considered a wide range of variables to allow a comprehensive understanding of the studied problem.

**Fig. 2:**
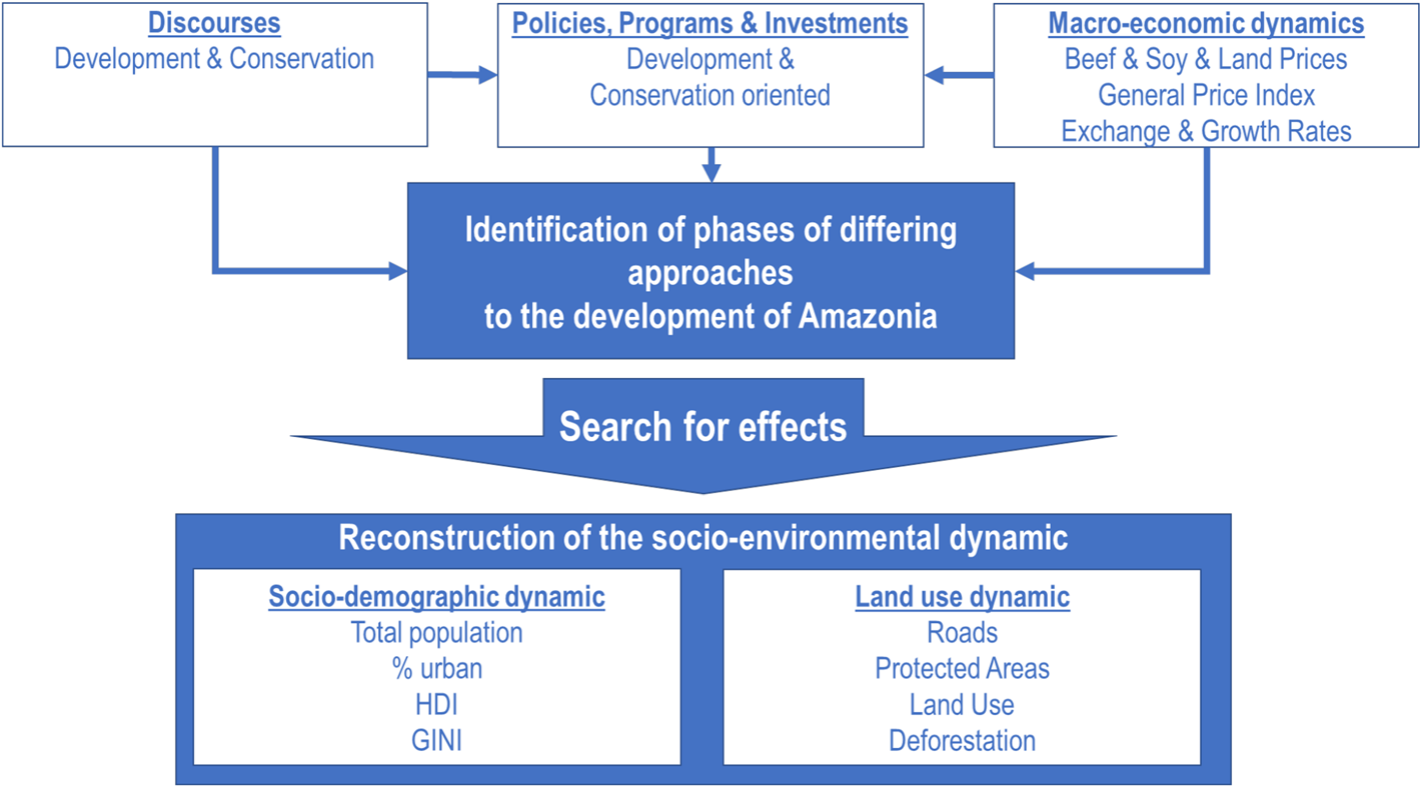
Analytical framework on the influence of discourses, policies, and macroeconomic dynamics on the socio-environmental dynamic in the Brazilian Amazon

Our study applied three analytical steps (Fig. 2). First, we traced timelines of the discourses, policies, programs and investments, and economic conditions that have been shaping the regional dynamic to differentiate phases with distinctive configurations of these five elements. Second, we computed the performance of socio-economic and environmental key indicators for the Brazilian Legal Amazon which includes the states Acre, Amapá, Amazonas, Pará, Rondônia, Roraima, Tocantins, Mato Grosso, and Maranhão. Finally, we compared the configurations of the factors of each phase with the average values and long-term trends of the socio-economic and environmental indicators to find evidence for causal dependencies.

### Definition of phases of Amazonian development

To identify phases with distinct configurations of discourses, policies, programs, investments, and economic conditions with relevance for the Amazon, we explored political and economic events and trends in Brazil but also globally. We considered presidential programs and policies, phases of economic booms and crises, and developments at the international level, and reviewed economic and conservation policies, and infrastructure investments into the region. We analyzed the relevant literature in international and Brazilian journals, pronouncements in political programs, project documentation, and statements by national and international economic organizations and the international cooperation community. We identified documentation relevant to our analysis using keyword searches in Google Scholar, Web of Science, and Scopus, as well as information on websites of international and national organizations and NGOs such as the World Bank, IFAD, FAO, IMF, BNDS, and IPUMPS.

To understand the changing economic conditions, we tracked changes over time of six economic indicators by exploring available statistics and reports. We expected global prices for beef and soy to have effects on investments in their production (Nepstad et al. 2006a,b). We recorded the fluctuation in exchange rates between the US dollar and the Brazilian currency, which influences the prices for export products but also agricultural inputs, and thus exports of the Brazilian agricultural sector. We analyzed the economic growth rate, which, when it increases, suggests a booming economy with high availability of capital for investment (Ross 2020). Data of these four indicators were obtained from World Bank data sets. Also, we observed land prices as an indicator of the availability of capital for agricultural investments and competition between investors and different land-use options (Sauer and Pereira Leite 2012). Finally, we considered fluctuations of the General Price Index (Índice Geral de Preços – Disponibilidade Interna (IGP-DI)) taken from the Fundação Getulio Vargas as an indicator of the general economic climate. A stable index correlates with a tendency for consolidation and long-term investments, while speculation and short-term investments, where land is prominently seen as a store of value, dominate during periods of a fluctuating GPI (Telles et al. 2016). Following an initial differentiation into phases, we searched for second-order policy changes (instruments to implement macroeconomic policies), and third-order policy changes (hierarchy of goals) (Hall 1993) to confirm the policy shifts between the outlined phases.

### Grasping the socio-environmental dynamic of the region

Following the definition of phases, we defined eight indicators suitable to capture socio-demographic and land-use changes in the period of analysis and during the outlined phases. Four indicators reflect socio-demographic dynamics: (1) Demographic growth results from natural birth and death, but it also indicates immigration into the Amazon region. This indicator reflects the attractiveness of the Amazon region to poor dwellers and results in the expansion of land occupation; (2) Increase in urbanization correlates positively with improved well-being due to income, access to electricity, sanitation, piped water, clean fuels for cooking, and heating, and reduced child malnutrition. The indicator also shows the abandonment of employment in agriculture as people seek employment in the industry and service sectors (Ritchie and Roser 2020); (3) The Human Development Index (HDI) combines information on income, health, and education, and its increase demonstrates socio-economic improvement (UNDP 2018); (4) The Gini Index measures trends in equity. A declining Gini Index concurring with an increased HDI reflects improvements for the poorer segments of society, whereas an increasing Gini Index and higher HDI suggest accumulation of wealth for a smaller portion of the population (Gini 1936).

Four indicators reveal changes in land-use dynamics and investments: (1) The construction of roads is the single major driver of forest conversion and land occupation (Novitski 1969; Fearnside et al. 2006), and thus is a proxy for land-use investments. Many roads in the Amazon are unpaved dirt roads, mostly constructed by timber companies, cattle ranchers, and mining companies. However, we only considered paved roads as the data on them are reliable partly because these roads are financed with public funds (Brandão Jr. and Souza Jr. 2006); (2) The demarcation of protected areas is commonly seen as an important measure to halt land-use expansion (Miller and Nakamura 2018). Accordingly, investments in protected areas indicate public interest in nature conservation. In Brazil, protected areas are organized in the National System of Conservation Units (SNUC) including several categories with varying objectives, functions, and instruments, administrated by federal, state, or municipal governments, or private owners; (3) The expansion of agricultural production land allows for several interpretations. First, it reflects the interests and the impact of land users who respond to discourses, policies, and macroeconomic conditions, more directly for example to prices and interest rates (Nepstad et al. 2006a,b), yet more indirectly to the climate of (un)certainty generated by the wider policy context (Telles et al. 2016). Second, it correlates with deforestation and environmental degradation, and, third, it demonstrates distributional effects associated with access to land. For instance, agro-industrial uses indicate the prominence of capitalized actors, while the expansion of pastures signifies a larger role of cattle ranchers, and mixed land uses are associated with smallholders (Chomitz et al. 2006); (4) The fourth indicator, deforestation rate, is inversed collinear with agricultural land expansion, but, if reducing, it also reflects success in the effort to conserve Amazonian forests (Angelsen 2010).

### Assessing the influence of shifting policy paradigms on the socio-environmental dynamics of the region

We plotted graphically the performance of each indicator for 50 years, or for the years for which data were available. This allowed a visual analysis of trends and changes, years of increase, decline, and stability on the course of time and during the outlined phases. In the case that indicator data were not available for each year, we completed the missing yearly values using simple linear interpolation (*lerp*). We then calculated and compared the yearly average change in each of the phases with the overall yearly average change during the 50 years. The calculated differences for each phase were expressed as a percentage deviation from the overall mean value. We classified the differences into five categories: much lower than the overall yearly average (˂ − 66%), lower (− 33% to − 65%), similar (− 32% to 32%), higher (33% to − 66%), and much higher (> 66%).

If there were no major differences in the performance of an indicator between a phase and the entire observation period, we assumed no effect. If we encountered accentuated differences, we looked for logical cause-and-effect relationships. In the case that we did find a plausible explanation for the observed changes in either the discourses, policies, or macroeconomic conditions, we took that as evidence of an effect. The consistencies for all indicators of a phase were then analyzed in the synopsis to find out which of the three factors (discourses, policies, and macroeconomic conditions) had the greatest explanatory value. Finally, the impact pathways suggested by the literature were examined for all phases to determine whether they were consistent and aligned over the entire period of analysis and whether and to what extent they represented long-term effects. Accordingly, repeated same-direction observations across the outlined phases, as well as the continuation of effects over several phases were taken as evidence of a strong influence of related discourses, policies, or macroeconomic conditions, respectively.

## Amazonian discourses: Policies–markets timelines

Until the pre-colonial era, the Amazon region was populated by a large diversity of indigenous groups. Some large groups managed surrounding environments to optimize the supply of forest, agricultural, and fish products (Clement et al. 2015). The unique biodiversity found in the region partly stems from thousands of years of indigenous land uses (Levis et al. 2012). The land-grabbing since the late sixteenth century by Europeans accompanied by murder, and illnesses, enslavement, and forceful relocation to settlements reduced heavily the original population. Only some of the indigenous groups survived and few have preserved their culture and original lifestyle (Moreira Neto 1988; Heck et al. 2005).

Since colonial times, products from the Amazon region were extracted to be traded in European markets. From the first half of the 17^th^ until the beginning of the eighteenth century, the transition of the economic life of the Amazon was caused by the extraction of spices and oils from forests known as *drogas do sertão* to be brought to Europe. Thousands of African slaves were taken to the region to complement Indian compulsory labor (Chambouleyron 2014). Around the same time, the domestication of native cacao (*Theobroma cacao* L.) began, reaching considerable economic importance until production shifted to the state of Bahia (Alden 1974; Homma 2014). Near major urban centers, landlords engaged in agriculture and cattle raising. In the nineteenth century, the Amazon region became the major provider of rubber for the rapidly growing industries in the United States of America and Europe, which brought immense wealth to the region (Bunker 1984). Again, thousands of families were brought to the region from all parts of South America and Europe to satisfy labor needs. These *golden times* (Weinstein 1983) abruptly stopped in the early twentieth century when rubber seedlings were smuggled to Southeast Asia, where they were intensively cultivated (Dean 1989). The worker families left behind were forced to find new ways of survival. They formed the so-called traditional communities that nowadays are scattered along rivers throughout the entire region (Lira and Chaves 2016).

After the early twentieth-century demise of the rubber boom, the Amazon region lost international economic relevance, and a prolonged period of economic stagnation followed (Godfrey and Browder 1996). Smaller booms followed: the expansion of Brazil nut production in southern Pará, gold rushes in several places (Cleary 1990), and a minor rubber boom revival during World War II (Araujo and Neves 2015). Access to the region’s road-free interior was difficult. Aside from spatially limited clearings along rivers, forests remained largely untouched (Kirby et al. 2006) until the late 1960s, when the region gained the interest of the military regime which recently had come to power and entered Brazilian geopolitics (Hecht and Cockburn 2010). Since then a continuous flow of economic actors has been entering the region in search of land, resources, and profits, or simply interested in making a better living. Contested frontiers emerged due to disputes over land and resources until then controlled by indigenous people and traditional communities (Schmink and Wood 1992; Simmons 2004).

**Fig. 3:**
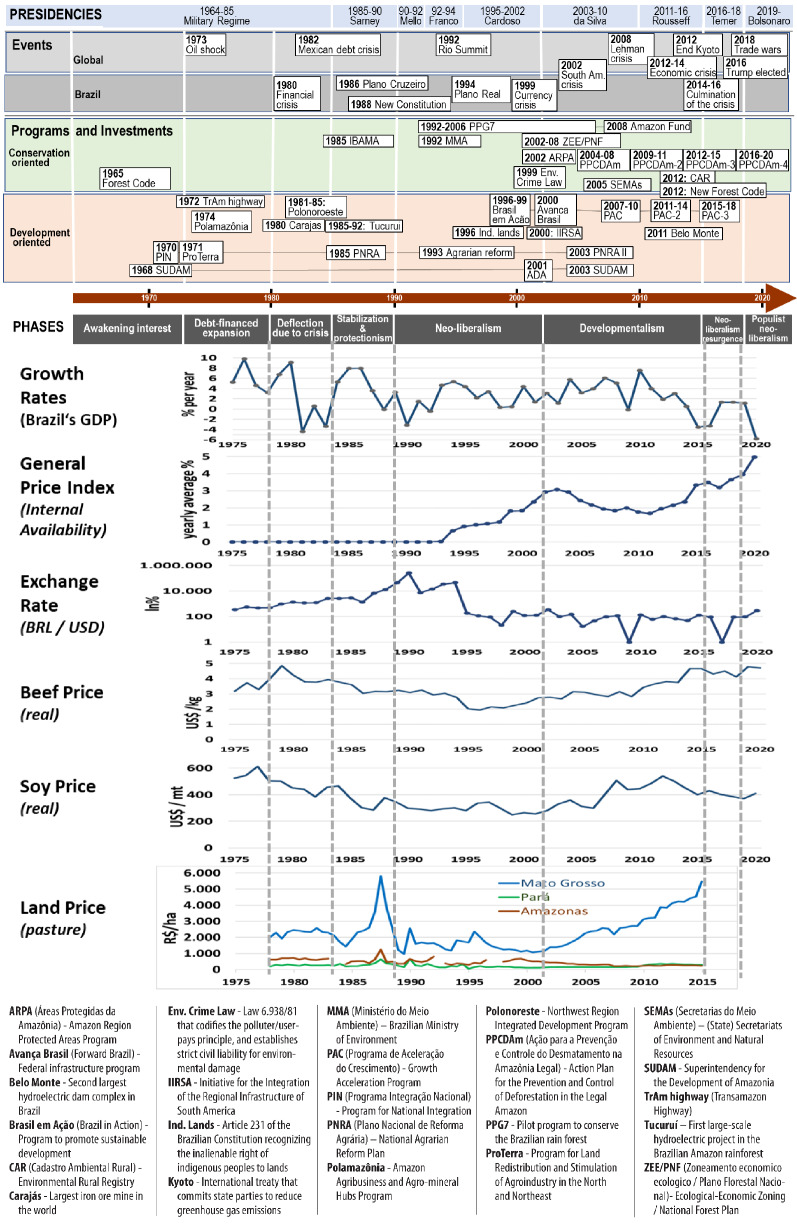
Eight development phases of the Brazilian Amazon and the performance of major economic indicators since 1970 (Data sources: The World Bank (beef & soy price, exchange & growth rate); Fundação Getulio Vargas (General Price Index & land price))

Discourses, policies, and economic conditions that have been shaping the Brazilian Amazon’s occupation have changed. Periods of economic growth and political stability interchanged with acute economic crises and political unrest (Fig. 3). Our analysis suggests the existence of eight major phases with distinct approaches to the development of the region: (1) Awakening interest (until the oil crises of 1973); (2) Debt-financed expansion (1974–1980); (3) Deflection due to crisis (1981–1985); (4) Stabilization and protectionism (1986–1990); (5) Neoliberalism (1991–2002); (6) Developmentalism (2003–2016); (7) Neoliberalism resurgence (2017–2018); and (8) Populist neoliberalism (since 2019). The first three phases correspond to the military regime period with dominant protectionist policies but little interest in market-oriented economic development. The last five phases correspond to an era in which neoliberal paradigms of economic development dominated public policies, with some variations across administrations.

### Phase 1: Awakening interest (until the oil crises of 1973)

In 1964, the Brazilian military took over control and formed a military government led by Castello Branco and Costa e Silva, which was consolidated during the Medici administration (1969–1974) (Codato 2006). The military government continued a previous import substitution model, but emphasized industrialization of iron and steel production, an increase of power generation, and road development, while also expanding agricultural and livestock production to improve domestic food supply (Andreas 2007). The neglected Amazon region came to the fore of the government with the *Operation Amazonia* program of 1966, which was summarized with the slogan ‘land without men for men without land’ (Hecht and Cockburn 2010). In addition to hoping to defuse land conflicts in other parts of the country through land distribution, the government wanted to occupy the areas along the national frontiers to secure geopolitical control over the region and its valuable resources (Kohlhepp 1984).

Accordingly, the military governments initiated programs to build roads into the Amazon to facilitate the expansion of commercial agriculture, extractive industries, and the settlement of colonists (Kohlhepp, 1991). Commercial agriculture was further supported with the allocation of public lands, fiscal incentives, and subsidized credits to incoming settlers. The colonization programs were carried out by the government but were implemented also with private involvement, particularly in Rondônia and Acre (Schneider 1995). The Superintendency for the Development of Amazonia (SUDAM) was created to attract national and international investments (Hecht 1985; Browder 1988; Binswanger 1991). Large development programs were initiated, partly financed by the World Bank, for example, the *Program for National Integration* (Plano de Integração Nacional-PIN) to promote colonization along the Trans-amazonian highway, and the *Program for Land Redistribution and Stimulation of Agroindustry in the North and Northeast* (ProTerra) to facilitate land occupation by middle and large landholders.

One of the few land-use regulations set up at that time was the revision of the Brazilian Forest Code (first introduced in 1934) mandating private landowners in the Amazon to maintain 50% of native forests on their properties as the so-called *legal reserves.*

### Phase 2: Debt-financed expansion (1974–1980)

The 1973 oil crisis caused severe economic turmoil. The Brazilian government started borrowing money from abroad to avoid the rise of domestic oil prices, and the ambitious investment programs of the early 1970s and during the Geisel administration (1974–1979) were financed with foreign capital (Hecht and Cockburn 2010). This resulted in a significant fiscal deficit. But, the policy of relying on external debt continued after the oil crisis to prolong the resulting boom (Coes 1995). The rationale was to pay the bill with revenues resulting from export-based economic growth and to keep debt interest rates low (Coes 1995).

During this phase, the overambitious original settlement programs were de facto halted, as the government had to realize that no large numbers of smallholders moved into the region. Other large-scale development projects sponsored by SUDAM (e.g., Ford, Volkswagen, Michelin) were also not particularly successful and attracted mostly medium-sized immigrant ranchers that settled mainly in Southern and Eastern Para (Pacheco 2009a). They benefitted from cheap land and credits with low interest rates to finance cattle ranching (Binswanger 1991). The Brazilian Agricultural Research Corporation (EMBRAPA) developed pasture varieties and cattle breeds adapted to Amazonian conditions (Serrão and Toledo 1990), and state agencies provided extension services to support smallholder commercial production of annual and perennial crops.

In 1974, the *Polamazonia* program was launched to stimulate extractive and agricultural activities through the creation of development poles for mining, logging, ranching, agriculture, and the production of hydro energy (Bunker 1994). The largest portion of the resources expended under this program went to three growth poles including the well-known Carajás iron deposits and the Tucuruí hydro dam (Mahar 1979).

### Phase 3: Deflection due to crisis (1981–1985)

In the late 1970s, the second oil crisis and the related global recession caused stagflation throughout the Americas. To stabilize the national economy, the *Figueiredo* government (1979–1985) devaluated the Brazilian currency, reduced export subsidies, and imposed price controls. With subsidized credits and low interest rates, the government promoted the agriculture and energy sectors. But, run-away inflation and economic slowdown turned into a recession, which, in combination with the need to continue servicing the external debt, caused a severe fiscal deficit (Coes 1995) and stopped foreign loans to Brazil since 1982 (Coes 1995).

As a result, it became increasingly difficult for the government to fund the expansion of infrastructure and growth poles. Prices of globally traded commodities dropped, which aggravated the fiscal imbalance. The government started printing money to continue financing development projects, resulting in chronic inflation and eventually hyperinflation. In response, it shifted its priority towards the stabilization of the national economy with drastic budget cuts and the protection of domestic markets.

When in Brazil unemployment reached dramatic heights, smallholders and the landless suffered particularly as a result of the promotion of industrial production of agricultural commodities, the Amazon became once again a region of massive social conflict. To calm the tense situation, in 1981, the government negotiated a US$ 1.6 thousand million loan for the implementation of the *Northwest Region Integrated Development Program* (Polonoroeste) seeking to pave 1500 km of roads and to resettle farmers, mainly from the state of Paraná, to Rondônia (Lele et al. 2000). This controversial project adversely impacted indigenous populations and forests (Fearnside 2005), which led the World Bank to suspend funding in 1985 (Kapur et al. 1997).

### Phase 4: Stabilization and protectionism (1986–1990)

In 1985, the military governments that had controlled Brazil for 20 years were replaced by the liberal-democratic Sarney regime. The new Constitution from 1988 transferred a large part of the federal governments’ revenues to the states and municipalities (Senado Federal 2016), and it recognized the rights of indigenous people to their traditional territories (Damasceno et al. 2017). Yet the country’s economy continued to face fiscal deficits, despite a positive trade balance, because of burdening debt payments (Coes 1995). In 1986, the government, pressured by the International Monetary Fund (IMF), adopted the *Cruzado Plan* to halt inflation and speculation, and to stabilize the national economy by introducing a new currency, freezing wages, and controlling prices. This, however, constrained domestic production, causing a scarcity of basic goods and the emergence of black markets (Coes 1995). To meet the domestic demand, imports became necessary, which again put pressure on the country’s fiscal reserves, which the government responded to with even more drastic measures to stabilize the economy.

The removal of subsidies to reduce public expenses particularly affected the Amazon, for instance when fiscal incentives and government credits to promote cattle ranching ceased in 1988 (Margulis 2004). By 1991, the government suspended all fiscal incentives for the establishment of new land uses (Schneider 1995). In the same year, income taxes for companies were lowered and interest rates increased. SUDAM financing continued, but it was also being investigated for corruption and political patronage (Pacheco 2005).

The increasing struggles over land led to the creation of the Extraordinary Ministry for Land Tenure Issues (*Ministério Extraordinário de Política Fundiária*) which had two regional offices in the Amazon (in Araguaia/Tocantins and Baixo Amazonas) to more effectively deal with high conflict areas. A process was started to differentiate public from private lands and to legally register public lands in the name of the Federal Government including future colonization target areas (Schmink and Wood 1992). In 1985, a national initiative to expropriate private landholdings guided by the Land Statute of 1964 was also applied to the Amazon (Fearnside 2001).

Since the mid-1980s, the conservation discourses of middle-class environmentalists, rubber, and indigenous movements converged and became connected to international discourses on biodiversity conservation, indigenous people’s rights, and climate change mitigation. Eventually, the Brazilian government adopted this pro-conservation discourse and explicitly acknowledged Brazil's responsibility to protect its forests (Banerjee et al. 2009).

### Phase 5: Neoliberalism (1991–2002)

In the climate of political instability in the early 1990s, the Collor (1990–1992) and Franco (1992–1994) administrations pursued classic neoliberal economic policies. To stabilize the national economy and to attract foreign capital, they restricted government expenses, raised domestic interest rates, and in 1994, introduced a new currency, the *Real,* coupled to a quasi-fixed US Dollar exchange rate. Subsequently, president Cardoso (1995–2002) privatized national state-owned companies. The reforms under this *Real Plan* kept inflation under control, assured the supply of cheap imported products, and stimulated a large inflow of foreign capital (Baer 2008), but worsened the fiscal deficit, which culminated during the 1998 Asia Crisis. Even then, the government continued its austerity package to keep stability, which by 1999 resulted in a fiscal surplus (Kaplan 2013).

Since the early 1990s, Brazil’s government tackled the issue of land distribution as a continuation of the land expropriation process initiated in 1985. A new agrarian reform gained momentum since 1993 particularly under the Cardoso administration (Fearnside 2001). Different from previous land allocation programs, the National Institute for Colonization and Agrarian Reform (INCRA) granted rights to landless people in the Amazon (Pacheco 2009b). Agrarian reform policies also included indigenous and traditional populations and their livelihoods as an alternative model of economic development (Vieira et al. 2014; Toledo et al. 2017). Additional efforts were made to identify and register federal public lands distinct from lands under states’ jurisdiction. Significant policy support was provided to social programs and renewed road expansion, for example, through the programs *Brazil in Action* launched in 1996 (IMF 2001) and *Advance Brazil* from 2000 (Nepstad et al. 2000). The *Regional Initiative for the Integration of the Regional Infrastructure of South America* (IIRSA) was launched to develop highway networks, river ways, hydroelectric dams, and telecommunications throughout South America with Brazilian leadership (Killeen 2007).

In this phase, also the environmental agenda gained momentum. In 1992, the UNCED in Rio de Janeiro consolidated a sustainable development agenda putting the spotlight on tropical deforestation, indigenous rights, and climate change (Pádua 2012). In its wake, environmental NGOs and organizations representing local forest users such as the *Coordinator of Indigenous Organizations of the Amazon River Basin* (COICA) emerged. The Brazilian government launched the ground-breaking *Pilot Program for the Conservation of Brazilian Rainforests* (PPG7) for the institutional strengthening of environmental agencies to support conservation and sustainable development projects, and to demarcate protected areas (MMA 2009). Key events were the creation of the *Amazon Region Protected Areas Program* (ARPA) (MMA 2020) and the demarcation of indigenous territories since 1996 (Presidencia da Republica 1996).

### Phase 6: Developmentalism (2003–2016)

The da Silva administration (2003–2010) modified the economic policies of the previous administration only slightly, despite its pronounced left-wing discourse (Barros 2017; Saad-Filho 2020). It pressed ahead with plans to consolidate Brazil’s role as a regional and global power. The *Brazilian Development Bank* (BNDES), already founded in 1952 to stimulate the expansion of industry and infrastructure in the country, and to support exports, technological innovation, sustainable socio-environmental development, and the modernization of public administration, became an important instrument of Brazilian Amazon policy, and significantly increased support for Amazonian agribusiness. The major beef corporation received aid to expand meatpacking plants in the Amazon, and support was provided for roads, ports, and trade facilities to create new export corridors for soy, beef, and palm oil. These plans were included in the *Growth Acceleration Program* (PAC) launched in 2007 (Ministerio do Planejamento 2010). In this phase, a stable global economy combined with rising prices for minerals and agricultural commodities generated impressive economic growth, also for the Brazilian Amazon.

The government combined policies aiming for aggressive national economic expansion with social and environmental policies. Support for traditional communities became mainstream although their implementation was less rigorous (Cano et al. 2015). The government increased minimum salaries, started social transfer payments to poor families (e.g., *Bolsa Família,* a program to provide aid to poor Brazilian families under the condition that their children attend school and are vaccinated), launched a land titling program to smallholders (*Terra Legal*), and invested in the provision of houses, electricity, and other public services (Ministerio do Planejamento 2010).

With the support of international cooperation, a 16-year *Action Plan for the Prevention and Control of Deforestation in the Legal Amazon* (PPCDAm) was launched to improve land tenure and territorial planning, boost environmental monitoring and control, and provide incentives for sustainable production. Parallel, environmental competencies were clarified and decentralized from the federal to the state level (Toni 2011). A revised *Forest Code* was adopted to more effectively address environmental crimes, and to foster sustainable forest management in private and public concessions (Amaral et al. 2017). The *Forest Code* made the *Environmental Rural Registry* (CAR) mandatory for rural properties. CAR called landowners, with or without legal land title, to report their property boundaries as well as the location of areas to be protected by law such as riverbanks, hillsides, mountaintops, and native forests. The purpose of CAR was threefold: to start clarifying unclear land tenure as a basis for resolving land conflicts; to effectively compel landowners to comply with their legal obligations for biodiversity protection; and to support landowners to improve land management and resource protection (Roitman et al. 2018).

By January 2020 over one million properties covering 220 million hectares had been recorded in the Amazon region. But, the implementation of the states’ *Environmental Regularization Programs* (PRA) to formalize the registration and oblige landowners to restore illegally deforested areas has been facing major challenges. CAR has been criticized for facilitating the seizure of land and the access to credit by capitalized actors at the expense of small landowners with poor access to information and communication media, and unable to comply with the environmental regulations (Godar et al. 2014; Jung et al. 2017; Assunção et al. 2020). The government’s environmental agenda has been questioned, because of the strong influence of the agribusiness lobby on the *Forest Code*, which caused a decline in environmental protection (Kröger 2020). Most strikingly, it legalized previous illegal land occupations and forest felling before 2008 and provided amnesty to the environmental debts of properties that would have been required to restore forests. It also contributed to an 80% reduction of permanent protection buffers along rivers and on hills and allowed the ‘restoration’ of natural habitats with non-native species.

By 2008, the government created the *Amazon Fund* to capture large international funds to finance avoided deforestation and forest conservation to contribute to climate change mitigation. Until 2020, the Amazon fund has received 1.29 thousand million US dollars from international donors (1.21 thousand million US dollars from Norway) for its demonstrated reductions in deforestation. It is now the globally largest results-based national funding mechanism for REDD + (Reducing Emissions from Deforestation and Forest Degradation and the role of conservation, sustainable management of forests, and enhancement of forest carbon stocks in developing countries). The Amazon Fund is managed by BNDES, the main financing agent for development in Brazil. Decisions concerning the implementation of the Amazon Fund at the BNDES are made by a Steering Committee, which consists of state, federal, and civil society actors exclusively from Brazil.

Growing business sector involvement was seen as critical for halting deforestation (Toledo et al. 2017), resulting in a soy moratorium, and the cattle agreements. The *Amazon Soy Moratorium*, signed in 2006, is an agreement by grain traders not to purchase soy grown on recently deforested land. It aimed to ensure that soy production in the Amazon region would only take place on existing agricultural lands and not result in new deforestation (Gibbs et al. 2015; Heilmayr et al. 2020). The cattle agreements were an outcome of high-profile campaigns by NGOs to stop meatpacking companies from purchasing cattle from properties with illegal deforestation. Companies signed the legally binding *Terms of Adjustment of Conduct* (Gibbs et al. 2016; Skidmore et al. 2020).

From 2000 to 2012, Brazil had one of the fastest-growing economies in the world. Prepared by the neoliberal adjustments of the *Real Plan*, Brazil was able to utilize its large reserves of raw materials coupled with low labor and production costs to profit from a commodity super-cycle induced by high demand for raw materials from the growing economies of China and India, and increased global liquidity induced by the 2008 financial crisis. However, with the decrease in commodity prices in 2011, the Brazilian economy began to falter and entered its longest recession (Wiggins et al. 2014; ECB 2016). This dynamic coincided with the start of the presidency of Dilma Rousseff, who was confronted with inflation that began to soar along with interest rates. Brazil entered a budget deficit, while the economy continued to shrink. Rousseff forced government-controlled banks and energy companies to keep interest rates low, cut taxes, and provide subsidized loans to certain domestic industries. Her government also imposed price controls on gasoline and electricity (Filho and De Paula 2015). These decisions during a time of political turmoil caused by scandal and corruption were not successful and resulted in the indebtedness of millions of middle-class families, reduced profits of public energy companies, and increased public debts (IMF 2016). This tumultuous context further weakened the conservation agenda. In an attempt to maintain her political power, Rousseff, after being closely elected for a second term, started to intensify collaboration with right-wing politicians representing the agribusiness lobby and large landowners and put them in charge of the Ministry of Agriculture.

### Phase 7: Neoliberalism resurgence (2017–2018)

After the collapse of the Brazilian economy and the impeachment of Rousseff, interim president Temer (2017–2018) changed the political course. He adopted pro-market policies, pushing for the enactment of new labor laws and the restructuring of pensions while repressing trade unions and social movements. In parallel, the government introduced austerity measures including significant cuts in public services, and the reduction in the number of *Bolsa Família* beneficiaries (De Oliveira 2018). Inflation and interest rates declined, and investments returned so that the national economy stabilized. But the massive cuts in the social budget increased extreme poverty and reversed the successes in the social arena of the previous years (IBGE 2019).

The government's new ultra-liberal economic approach significantly improved conditions for large-scale rural investment in the Amazon region (Tollefson 2016). It even threatened the integrity of protected areas by facilitating access to mining and agribusiness companies (Agencia Brasil 2017). Several initiatives of the government attempted to weaken the legal constraints for infrastructure licenses such as dams, roads, and agricultural projects.

The erosion of social and environmental policies by the previous governments, combined with corruption scandals, sparked concerns (Rochedo et al. 2018) and mass protests against Temer (Tollefson 2016). His refusal to resign made him increasingly unpopular and provoked a general atmosphere of uncertainty, plunging the country into crisis and its worst recession in history.

### Phase 8: Populist neoliberalism (since 2019)

In January 2019, the right-wing populist Bolsonaro assumed the presidency because many Brazilians disapproved of status quo politics and voted for a political change. His administration, strongly supported by evangelical groups and the military, embarked on a dismantling of social and environmental achievements of former neoliberal governments, from Cardoso to da Silva and Rousseff. Bolsonaro favored the private sector as the motor of economic growth, further reduced social programs, education, and health, and promoted a law-and-order approach ostensibly to end years of corruption and spiraling gang violence. His populist hardline, anti-establishment proposals had difficulties getting approved by Congress. However, his administration was successful in overhauling the country’s pension system that accounted for 40% of total federal spending. Other measures remain pending including ambitious tax reforms, curbing public spending, and selling off state companies (MacDonald 2020). At the country level, there had been indications of a slow recovery of the national economy after six years of negative and stagnant growth. However, the political uncertainty under Bolsonaro provoked a capital flight and accelerated a significant currency devaluation in 2020 (Oyamada and Batista 2020). In parallel, the economic situation has been seriously affected by an out-of-control spread of Covid-19 partly caused by political mismanagement (Pasquali 2020).

Concerning the Amazon, the discourse of the Bolsonaro administration represented a drastic shift in comparison to the former phases. He denies the existence of climate change and explicitly promotes economic development at the cost of forests. He loosened regulations on land ownership making it possible for land grabbers to simply self-declare their ownership of a given piece of territory. He reduced indigenous’ rights (Carvalho et al. 2021) and encouraged the commercial exploitation of their protected lands with the pretended argument that Indigenous People also aspire to progress and economic well-being (Fellet 2020). The Environment Minister Salles (2019–2021) legally charged with malfeasance partly because of his dealings in the Amazon, pushed through further deregulation of environmental policy, including a drastic reduction of environmental fines (Spring and Eisenhammer 2019) and the dismantling of environmental agencies and budget cuts of IBAMA, the main state agency in charge of environmental control (Escobar 2019). This together seriously affected environmental law enforcement and increased environmental law violations (Carvalho et al. 2019).

## Socio-environmental dynamics in phases of changing policy and economic conditions

Over the last five decades, discourses, policies, and economic conditions have changed in Brazil, sometimes with great pace. Despite a certain degree of political and institutional coherence, each of the eight development phases summarized above has its unique features and socio-environmental trends. This section reviews the performance over time of indicators of socio-demographic and land-use dynamics during the eight phases.

### Socio-demographic dynamics

**Fig. 4:**
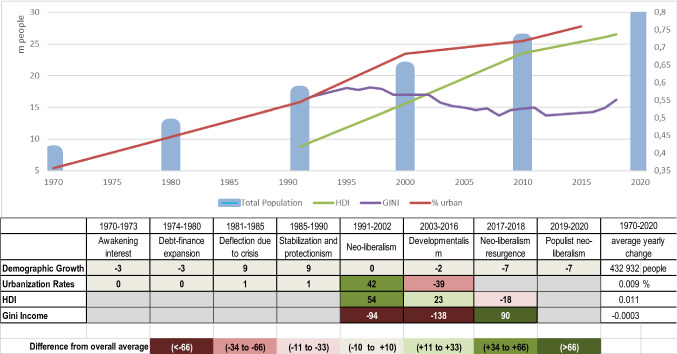
Dynamic of key socio-economic indicators for Brazil’s Legal Amazon since 1970, and percentage deviations of the yearly average change in each development phase from the overall yearly mean during the observation period (Data sources: IBGE Demographic Census (total population, GINI, % urban); Global Data Lab and Human Development Atlas (HDI))

Figure 4 shows the performance since 1970 of (1) demographic growth, (2) urbanization rates, (3) the Human Development Index (HDI), and (4) the Gini income index.

Demographic growth follows a nearly linear trend with a slight slowdown since 2017. On average, the population in the Amazon increased every year by more than 430 000 people. Urbanization rates increased, particularly during the Neoliberal phase between 1991 and 2002. The HDI steadily increased but this increase was less pronounced since 2010. This is not the case with income distribution. In contrast to the generally positive linear trend of the previous three indicators, the Gini Index showed much stronger changes over the last 50 years. It declined drastically between the mid-1990s and 2008, but since 2016 increased again.

The steady demographic growth indicates that since the first government’s efforts to settle people in the region during the initial stages of the Brazilian Amazon occupation, the flow of immigrants kept growing, to both rural and urban areas. The creation of job and income opportunities linked to road construction, the building of hydro dams, as well as the development of mining operations are factors that explain the migration streams (Lisansky 1990). Despite changes in infrastructure investments, over time and between locations, it appears that the factors that attract people to the region remain constant, and are independent of the discourses, policies, and economic trends considered here.

Similar to population, urbanization rates have increased steadily since the 1970s. Since cities began to expand, many residents of rural areas moved to cities where they joined more recent extra-regional immigrants, in search of better education, access to other services, and paid jobs (Godfrey and Browder 1996). People did not only relocate to the two Amazonian metropolises Belém and Manaus but also to many smaller urban centers, which led to the emergence of sub-regional urban–rural networks characterized by economic and political inter-dependencies, population movement, and provision of services (Guedes et al. 2009). The strongest increase in urbanization rates happened during the Neoliberal phase, whereas during the Lula da Silva administration, rates declined which reflects a reduced rural–urban flow likely caused by policies to improve the well-being of rural residents. At present, more than three-quarters of the Amazonian population is living in cities. Since the early 1990s, the total rural population in the legal Amazon has not been growing anymore.^1^

The steadiness of the increase in the HDI over the past 50 years suggests that no specific measure in one of the phases has influenced this trend. The slightly reduced increase of the HDI in the Developmentalism phase coincides with the end of the commodity boom in 2012. However, much of the improvements reflected in the increased HDI are to the benefit of the ever-increasing urban population. Much of the rural population still have poor access to quality healthcare and education and lives in poverty (Guedes et al. 2012). The data even suggest that a decline in the prosperity of the region’s rural population has been aggravated by the policies of the recent two presidencies.

Between 1991 and 2016, the Gini Index significantly improved indicating an equity effect of the policies of the Neoliberal and Developmentalism phases, particularly during the Lula administration, which emphasized social policies. The positive performance of the Gini Index also coincided with the positive economic climate during those years and the emergence of a larger middle class in the cities (Wogart 2010). The positive Gini Index trend abruptly ended with the economic crisis in 2012. Since 2017, the changes in the Gini Index have been stronger than the changes in the HDI. This coincides with the reorientation of economic and social assistance policies during the right-wing governments of Temer and Bolsonaro, which dismantled social safeguards and promoted the interests of economic elites (Webber 2020).

The above analysis allows for two major conclusions. First, the wider demographic and urbanization trends appear uncorrelated to the discourses and policies of different phases. There is a constant inflow of people into the region and progressively a larger part of the Amazonian population decides to live in the region’s metropolis and urban centers. Second, there is an influence of policies and economic trends on well-being and wealth distribution. Well-being performance development concurs with the economic development of the region, and the Gini Index is sensitive to policies. The socially oriented policies in the phases of the 1990s and 2000s, reflected in social justice discourses, were replaced by phases focused on promoting the interests of elites, as was already the case in the 1980s.

### Land-use and dynamics

**Fig. 5:**
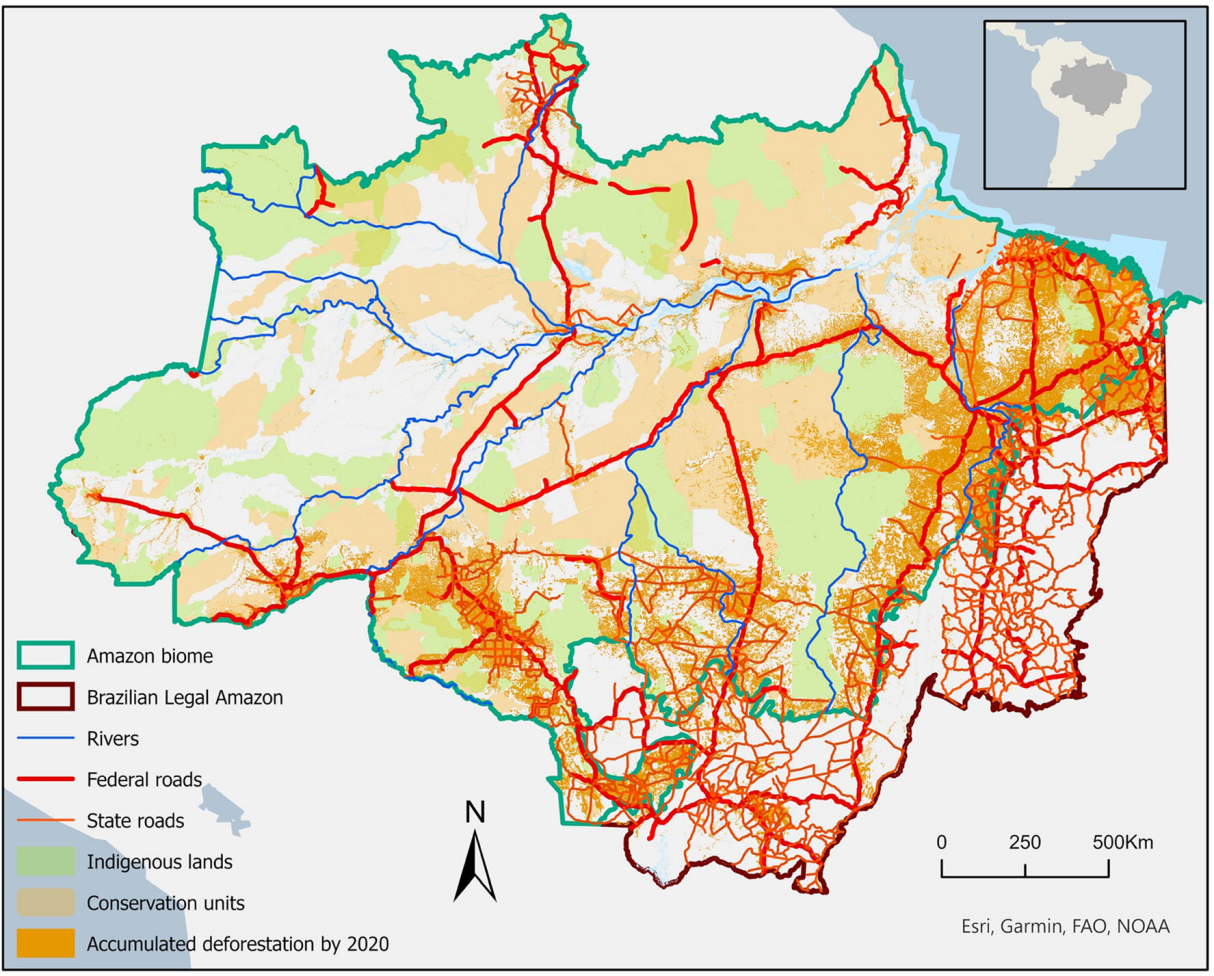
The current state of roads, protected areas, indigenous lands, and deforestation in the Amazon biome (Data sources: INPE TerraBrasilis (deforestation); Brazilian Ministry of Environment (MMA) (conservation units); FUNAI (indigenous lands); Sistema Nacional de Viação (roads))

This section analyses the changes of the four land-use indicators: road construction, demarcation of protected areas, land-use change, and deforestation to assess the environmental outcomes of discourses, policies, and economic trends (Fig. 5).

#### Development of roads and protected areas

**Fig. 6:**
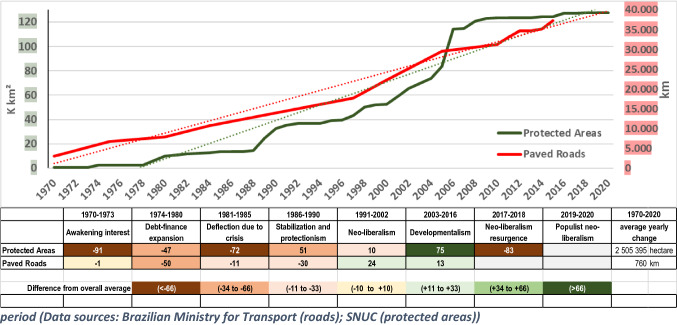
Expansion of the length of paved roads and Protected Areas in Brazil’s Legal Amazon, and percentage deviations of the yearly average change in each development phase from the overall yearly mean during the observation period (Data sources: Brazilian Ministry for Transport (roads); SNUC (protected areas))

Figure 6 shows, as proxies for investments in economic development and nature protection, the length of paved roads and the area classified as protected areas in Brazil’s Legal Amazon since 1970.

The length of paved roads in the Amazon region has been steadily increasing since 1970. Construction of roads started in the late 1960s with the Brasilia-Belem highway, followed by the Trans-Amazonian highway in early 1970s. Until the end of the fourth phase, Stabilization and Protectionism, the average annual increase was 760 km. Since the start of the neoliberal phase in 1991, the road network increased at a higher pace, but again fairly constantly, with modest variations. The road expansion, however, was not influenced much by variations in Amazon development interventions of the different phases. Public infrastructure investment policies became prominent during the 2000–2010 period, with the launching of the *Growth Acceleration Program* (PAC) in 2007 (Santana and Nascimento 2012). Variations in road construction programs reflected the vagaries of the economic situation of the country. Accordingly, since the economic decline of 2012, the construction of roads has been slowing down.

Protected areas equally show a significant increase since the 1970s. Nowadays, the Legal Amazon holds 118 million hectares of classified protected areas. The first protected areas were established towards the end of the 1960s when the military government began its Amazon integration policies. Since then, protected areas have been demarcated inthree phases from 1986 to 2016 with a minor peak around 1990 that coincided with the preparation years of the UNCED held in 1992, and a marked increase during the Lula da Silva administration. Since 1995, the demarcation of protected areas was explicitly linked to the recognition of the rights of traditional communities and indigenous peoples to land and resources (Benyishay et al. 2017; Reydon and Fernandes 2017). Later, the demarcation of protected areas dedicated to sustainable exploitation gained importance as a model for sustainable local development, particularly in the form of extractive reserves for traditional communities (Toledo et al. 2017). Since 2008, nearly no new protected areas have been demarcated, and some have even been downsized. This coincided with the start of the then global financial crisis, but may also reflect a shift to a new nature conservation thinking to let market forces play a larger role in nature conservation (Godar et al. 2014; Toledo et al. 2017). Since the Temer administration, there has been an explicit departure from protected areas as the main thrust of government nature conservation policies, and Bolsonaro’s disregard for environmental crimes has led to a de facto lifting of protected area status in many places (Carvalho et al. 2019).

#### Area under production

**Fig. 7:**
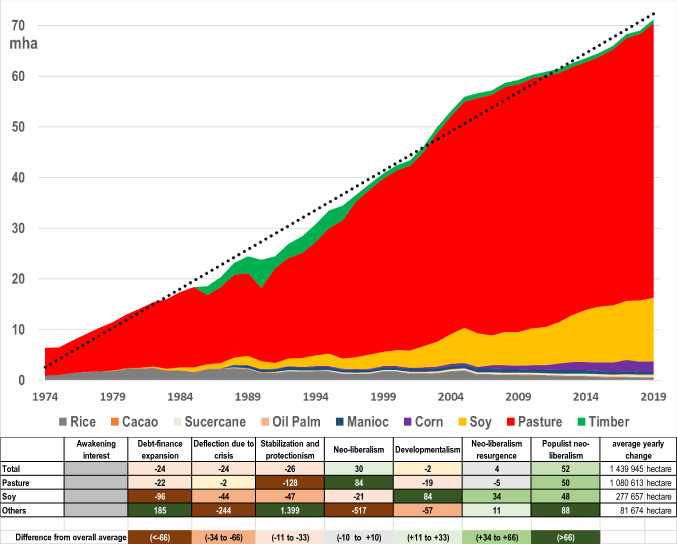
Area of production related to the principle agricultural products and timber in the Legal Amazon, and percentage deviations of the yearly average change in each development phase from the overall yearly mean during the observation period (Data sources: SIDRA (land uses except pasture); MapBioma (pasture 1985 to 2019) and Agrarian Census (pasture 1975 to 1980))

Since the opening of the Brasilia-Belem highway, the area under agricultural production has been increasing continuously. Figure 7 shows how much land in Brazil’s Legal Amazon was designated for the production of nine key commodities from 1974 to 2016.

Figure 7 shows a continuous expansion of the area under production in the Legal Amazon. Until 1990, yearly expansion was below the overall average. Then, in the phase of Neoliberalism, characterized by large infrastructural investments, settlements, and the liberalization of markets, the expansion of the production area grew sharply until around 2005. Since then, the pace of expansion has dropped to the level before 1990.

Over the last 50 years, 75% of land occupation in the Amazon has been done by extensive cattle ranching. Already in the 1970s, the area of land used for cattle ranching was significant. Converting forestland to pastures was the fastest and most effective option to establish land ownership (Faminow 1997). Cheap land and easy credits made cattle ranching expansion attractive until the mid-1980s (Hecht 1988). Then, the pace of expansion of pasture lands declined as a result of the austerity programs during the Stabilization and Protectionism phase, during which fiscal incentives were cut. In the subsequent Neoliberalism phase, cattle ranching expanded again in response to public investments in infrastructure and processing facilities, which made cattle ranching profitable without direct subsidies (Margulis 2004). The investments into processing facilities, slaughterhouses, and storage facilities closer to the production zones have been continued and even intensified until today (Pacheco and Poccard-Chapuis 2012). In the subsequent phases, other factors became driving forces such as the devaluation of the Brazilian currency which led to a growth in Brazilian beef exports, and successful efforts to control livestock diseases (Kaimowitz et al. 2004). Since then, cattle ranching expansion oscillates between 1 and 2 million hectares a year, which might signal a limited capacity among Amazonian farmers and urban investors who are becoming increasingly relevant for this expansion (Serra 2020). There is also a long-term trend of cattle management intensification which allowed the grazing capacity to increase from 0.69 heads hectare^−1^ in the 1990s to 1.56 in 2012 (Dias et al. 2016). Years with lower pasture expansion rates reflect a general decline in economic activities and a decline in land speculation in times of crises since 2008 (Brito et al. 2019). Nevertheless, the forestland grabbing by ranchers remains high and has again gained momentum during Bolsonaro’s administration (Carvalho et al. 2019), concurring with significant increase in forest fires (Cardil et al. 2020).

Since the Neoliberal phase, soybean production has been expanding. Agro-industrial production of soy requires high investments available only to capitalized actors (Richards and Arima 2018). Particularly, during the neoliberal phase, soybean became the second dominant land use in the region. First limited to the state of Mato Grosso, genetic and technical improvements of soy germplasm allowed its expansion into the wider Amazonian territory (Kaimowitz and Smith 2001). The expansion of soy had been explicitly favored by all governments since the Cardoso administration (1995–2002), and the support for agro-industries has been growing since then, not only in Brazil (Garrett et al. 2018; Kröger and Nygren 2020). Since the Developmentalism phase, massive investments into improved storage facilities and logistics for grains have been launched (Rausch et al. 2019). This process has been accompanied by a dramatic increase in the level of foreign investment in land and agriculture for the production of commodities, which was also reflected in the sharp increase in land prices and concentration (Sauer and Pereira Leite 2012). Much of the expansion of soy, particularly in Mato Grosso, took place on former pasture land, which has possibly resulted in a displacement of cattle ranching to new agricultural forest frontiers, particularly in the State of Para (Arima et al. 2011). An even stronger encroachment of soy production into forest areas in the Amazon might have been avoided by the soy moratorium, an example of market-based conservation (Gibbs et al. 2015; Heilmayr et al. 2020).

In comparison to cattle and soy, the production of other crops and timber plays only a marginal role in terms of areal expansion. Only governments that promoted early colonization supported the cultivation of a larger range of annual and perennial crops. But the effects of many programs to promote more diversified agricultural production remain low (Pokorny 2013; Barbosa 2015). Recently, there has been increased support for growing cocoa in agroforestry systems, but cultivated areas remain still comparatively small.

Since 1970, land use in the Amazon has expanded constantly, demonstrating periods of greater and smaller increases. Land use expanded independently from oscillating prices for beef and soy, or other macroeconomic parameters. Differences in development and conservation policies and private sector investments of the eight phases, however, played a role in several ways, such as the provision of fiscal incentives for cattle ranching in the initial stages of colonization, and the investments in road building and processing facilities for soy and beef since the beginning of the Neoliberalism phase in the 1990s. The dominance of cattle and the gradually increasing production of soy on most suitable previous pasture lands could be interpreted as policy outcomes. But we found mostly evidence of policy effects stimulating livestock and soybean expansion, but only a little evidence of improved environmental policies and regulations since the 1990s that reduced their production and negative environmental impacts. Periods of reduced land-use expansion, such as at the end of the Developmentalism phase, lasted only for short times. Nor did technological progress that contributed to land-use intensification result in any lasting decline of land-use expansion.

#### Deforestation

**Fig. 8:**
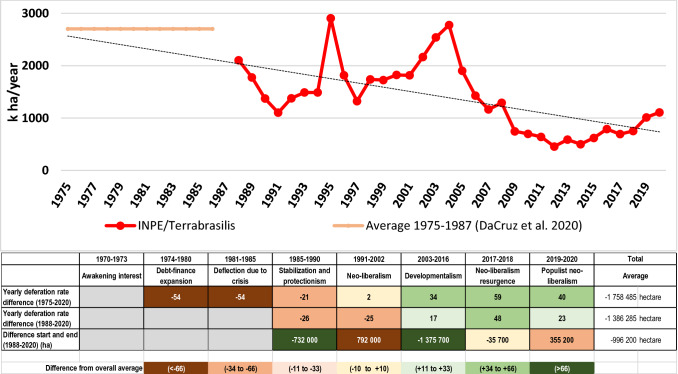
Annual gross deforestation within the Legal Brazilian Amazon since 1975, and percentage deviations of the yearly average change in each development phase from the overall yearly mean during the observation period (Data sources: INPE/Terrabrasilis (1988–2020); Da Cruz et al. 2021 (1975–1987)

Since the large infrastructural expansion programs of the late 1960s, the Amazon region has been subject to fluctuating but constant deforestation (Fig. 8). On average, nearly 1.8 million hectares of natural forests were destroyed annually. But, the historical gross deforestation rates since 1975 show a steady decline from the first phase of colonization until today, however, with distinct phases of higher and lower deforestation. Historical peaks were 1995 and 2004, and there was a significant decline from 2005 to 2012 since when it has been steadily increasing again. Annual deforestation has surpassed again the one million hectares mark in 2019/2020. The long-term view shows that despite an overall decrease in yearly deforestation, especially after the 2004 peak, periods of lower deforestation were always followed by periods of higher deforestation, and vice versa.

The eight development phases reveal strong differences in deforestation. The high deforestation rates during the 1980s correspond with the large investment programs of the military regime. Only during the phase of Stabilization and Protectionism, did deforestation rates decline, corresponding with the massive reduction of state-financed development programs. The accelerated increase in deforestation at the beginning of the subsequent Neoliberalism phase as well as the 2004 peak both coincided with strong currency devaluations (Kaimowitz et al. 2004), but also with the announcements of stricter environmental legislation by the respective governments. In this respect, these peaks could also be the result of attempts by land users and speculators to secure their interests before the announced restrictions come into force. The subsequent reduction of gross deforestation after these peaks supports this interpretation. The average annual deforestation rates of the Neoliberalism and Developmentalism phases were below the overall yearly mean of the whole period, but this trend reversed during the Neoliberal resurgence phase. This more recent increase in deforestation suggests that it may be associated with a weakening of the environmental regulations and low enforcement capacity.

The longer periods of reduced deforestation strongly coincided with phases of severe financial crises and economic instability, and with reduced availability of capital and related subdued interest to invest in agricultural production (Wunder 2001). The low deforestation of 1997 has also been attributed to declining prices of export commodities, such as soy and beef (Fearnside 2017). But, the decline in deforestation between 2005 and 2012, during the Developmentalism phase coincided with attractive agricultural output prices, which, in theory, should have stimulated investments. This suggests that other forces played a role during this period.

The reduction of annual deforestation during the 1990s and the massive slowdown after 2005 has been labeled as an achievement of well-designed environmental instruments and institutions (Macedo 2012) and the increased spending on environmental control (Assunção and Gandour 2015). Some authors claim that these demonstrate the benefits of private sector-led conservation efforts that were aided by progress in public property registers and deforestation monitoring (Heilmayr et al. 2020). But, the sharp re-increase in deforestation after the millennium as well as in recent years also demonstrates the challenge to keep measures that contribute to reducing deforestation in place and effective in the long run. The increasing trend of deforestation since 2012, which is more accentuated since 2017, indicates a lack of a long-term effect even of the celebrated conservation policies of earlier years.

The end of Developmentalism saw a drastic departure from these policies and a resurgence of neoliberalism-inspired policies. Particularly, the anti-forest and anti-indigenous neoliberal populist discourses and policies of the Bolsonaro administration and the taking advantage of this opportunity by investors caused the deforestation upsurge, exacerbated by forest fires linked to climate change (Artaxo 2019; Carvalho et al. 2019; Pereira et al. 2020). Land speculation may also play a significant role in triggering land occupation and deforestation driven by the expectation of growing land values (Miranda et al. 2019). It is estimated that in this period alone about 17% of the original forest area in the Amazon region has suffered degradation (Bullock et al. 2020). However, despite this deforestation upsurge, deforestation in 2020 was still 40% lower than the 45-year average. But, the recent drastic increase in deforestation is alarming and erases much of the deforestation reduction since 2005.

## Discussion

### Constants and changes in discourses and policies

The analysis above demonstrates both continuity and change in discourses and policies related to the Brazilian Amazon during the last 50 years. Those that aimed to assure sovereignty over the national territory and to facilitate economic use of land and natural resources persist until today, although social and environmental concerns have also become part of the mainstream policy, yet with different attention in the last four phases. The military governments pursued Amazonian development policies inspired by a national protectionism doctrine, whereas the following democratically elected governments adopted variants of neoliberal doctrines and policies. The lack of a development approach with a specific Amazon perspective has been a consistent element over the years. From the beginning, policies were mainly driven by concerns and influence from outside. In a certain way, also the regular occurrence of economic and political crises (1980, 1999, 2008, 2012, see Fig. 3), economic booms, and periods of stagnation were consistent features for the last fifty years. Specific policy responses to crises varied with an emphasis on either public investment or austerity programs.

In contrast to this, the environmental and social agendas have changed profoundly over time. The democratically elected neoliberal administrations moved from just embracing economic goals to mainstreaming social and environmental concerns, in line with international discourses and political agendas, and relying largely on conditional cash transfers and market-based mechanisms. Over the years, also the complexity and diversity of the political landscape increased. Efforts were made to balance economic growth, nature conservation, and social goals, and to create decentralized administrative structures as a basis for more effective regional governance, applying regulatory area-based approaches (e.g., setting aside protected areas, zoning, moratoria) and promoting social agreements to help to enforce land-use regulations. Nonetheless, the recent emergence of populist neoliberalism ultimately points at a revival of the Wild West discourse prevalent among the military governments of the 1970s.

### Irreversible quantitative development effects, but scope for influencing social equity

Concerning the analyzed quantitative development indicators, we saw a constant, often even linear long-term trend, despite some ups and downs during the eight phases. Most constantly was the regional population growth and urbanization increase, with a somewhat under-average increase during the Developmentalism phase (2003–2016). The land brought under agricultural production also expanded constantly with a long-term average yearly increase of nearly 1.5 million hectares. The expansion was strongest during the 1990s Neoliberal phases despite the parallel establishment of nature conservation goals in the political agenda. Also, significant deforestation peaks fell within this area, but at the same time, environmental policies and governance arrangements during the Neoliberal phases resulted in deforestation rates that are well below the yearly mean for the period 1970–2020. Recently, with the populist right-wing governments since 2017, land-use expansion and deforestation have slightly accelerated compared to previous years. Since the 1970s, land-use expansion principally was linked to the cattle economy leading to a conversion of forests into pasture land, with the exception during the Stabilization and Protectionism phase at the end of the 1980s. Consistent with this has been the expansion of the road network that showed an accentuated growth since the 1990s Neoliberal phase. These constant infrastructures, land use, and demographic trends could be interpreted as effects of two interventions reflecting the economic growth discourse: First, the colonization programs of the 1970s that were the start of a persistent land occupation process (Kirby et al. 2006), and, second, the promotion of agriculture as the driver of the region’s economic development, particular cattle production (Pacheco 2009a), and later also soybean cultivation (Garret et al. 2018). Most of the other differently accented development and conservation policies sometimes showed effects which, however, were seldom sustainable. Particularly, the progressive increase in social equity indicates an effect of the social development programs that started in the late 1990s and continued during the Neoliberal and Developmentalism phases (Guedes et al. 2012). These programs, however, turned out not sustainable, because the anti-social policies of Temer and Bolsonaro managed to reverse positive trends in only a short time (IBGE 2019).

### No satisfactory long-term effects of environmental policies

Similarly fragile appear the outcomes of the efforts to protect Amazonian forests and their indigenous and traditional populations, although the constant nearly linear expansion of land under agriculture has been accompanied by an overall decrease in deforestation rates, especially during the Stabilization and Protectionism phase at the end of the 1980s and the Developmentalism phase during the 2000s. However, even during the phases when environmental protection became increasingly important for national and international agendas, land use consistently expanded and deforestation fluctuated widely. This is particularly obvious in the Neoliberalism phase, following the UNCED 1992, as well as during the Developmentalism phase in the early 2000s, when the outspoken and influential Minister of Environment, Marina Silva, pushed forward a series of environmental reforms (Barbosa 2015). The limited effectiveness of environmental policy commitments is evidenced by the historical 2004 deforestation peak of three million hectares during exactly this phase. The subsequent drop of deforestation rates to a minimum in 2012, however, could indeed be interpreted as a long-term policy effect. But deforestation rates have nearly tripled since then and accelerated during Bolsonaro’s presidency, which does not support the idea of lasting impacts of such environmental policies.

The case of protected areas also exemplifies the lack of long-term outcomes of conservation policies. The establishment of indigenous lands in the 1990s and protected areas for sustainable use in the early 2000s reflect the success of efforts to halt the persistent expansion of agriculture into forestlands and its negative impacts on indigenous and traditional forest groups (Toledo et al. 2017). Yet, despite evidence suggesting the positive conservation effects of these policies, particularly of the indigenous territories (Porter-Bolland et al. 2012; Nolte et al. 2013; Schuster et al. 2019), the measures had limited conservation effects at a regional scale because encroachment of these areas has continued or moved to less protected areas (Ribeiro et al. 2018). Furthermore, since then, the state has been reluctant to grant further tenure rights to traditional and indigenous groups, and, as observed, the new demarcation of protected areas has even completely stagnated since 2005.

Deforestation has remained high in the Brazilian Amazon, even during years of declining deforestation rates. This indicates a structural dominance of environmentally adverse development interests over proliferating conservation discourses and policies. However, not all beef and agricultural production in the Amazon is associated with deforestation due to growing yields in already converted lands. While there has been a partial delinking between agricultural production and deforestation, deforestation is still driven by agricultural expansion and land speculation (Brito et al. 2019). This structural problem is reflected in other sustainable agriculture initiatives that are aimed at facilitating cattle ranchers and agribusiness entrepreneurs to produce without deforestation (Brannstrom 2009; Nepstad et al. 2014). Our analysis confirms that such policies can have positive effects. Even if partially reduced by the latest developments, these achievements will also benefit future generations, not just because of the emissions from deforestation that were avoided over the last twenty years, the release of which would have even more reduced our chances to achieve international climate goals. However, it remains unclear whether and to what degree these successes are durable and sustainable, especially when considering the possibility of leakage (Toledo et al. 2017; Garrett et al. 2018). On the contrary, cattle ranching continues to be the main driver of deforestation in new frontier lands, and land-use dynamics accelerated again in recent years (Carvalho et al. 2019) as happened during the 1980s (Kröger 2017).

### Diffuse influence of macroeconomic conditions

Our analysis confirmed the effect of changing macroeconomic conditions. Particularly, in times of economic crises, the reduced availability of capital for investments, but also a declining expectation for short-term profits among entrepreneurs from large cattle ranchers and agro-industries dampened land-use dynamics (Fearnside 2017). This crisis effect, however, was always only temporary, also because during such periods’ governments responded by offering attractive investment conditions to domestic and foreign entrepreneurs, or funding and subsidizing infrastructure investments (Banerjee et al. 2009; Garrett et al. 2018). Crises can also amplify land-use dynamics, e.g., when production area expansion is used as a strategy to mitigate declining export revenues (Mueller and Mueller 2016; Arias et al. 2017). The last two Brazilian presidents promoted again policies to exploit Amazonian resources to achieve economic growth and to counteract the tense social situation caused by national crises (Souza and Hoff 2019; Webber 2020). This also indicates a limited effect of the fluctuating commodity prices since the 2000s global boom on Amazonian land-use expansion. Land-use expansion in the Amazon appears to have become independent from such market signals. In addition, this may be so because land constitutes the most precious commodity in the long run given expectations of profits.

## Final considerations

Our analysis revealed for many of the variables examined continuous, often even linear long-term trends. But, there were also some changes and fluctuations that indicate that policy actions can generate environmental, social, and economic improvements. However, most of the observed policy effects were short-term, spatially limited, or simply insufficient. Periods of accelerated land-use expansion were followed by a decline in activities requiring new lands, and vice versa. This observation challenges the interpretation that the decline in deforestation rates by 2012 represented a breakthrough in national and international efforts to combat deforestation (e.g., Scholz and Schöneberg 2007; Macedo et al. 2012; Nepstad et al. 2014; GIZ 2015; Gibbs et al. 2016; Assunção et al. 2020; Stabile et al. 2020). Our analysis suggests the limited validity of such conclusions and points at the importance of a historical and regionwide perspective. We expect that a comparison with other regions would have corroborated these findings (Ahram et al. 2018).

The reduced long-term conservation effect of environmental discourses and policies, and market signals indicates a structural dominance of economic development concerns. The original mindsets of the military government to assure territorial integrity and mobilize the Amazon region’s rich resources for national economic growth and people’s well-being proved to be difficult to reverse. Individual aspirations to satisfy basic needs and to achieve material prosperity in combination with a collective competitive world view and persistent power imbalances (Farias Filho 2011) grounded in a history of clientelism, oppression, and marginalization of the local (Pokorny 2013), converged into a unidirectional force and became a rigid growth dynamic generating undesirable outcomes to forests and its populations (Ward et al. 2016; Wiedmann et al. 2020). Our analysis suggests that this force is difficult to control or govern with environmental protection and sectoral sustainable development policies. Accordingly, expectations of ongoing initiatives for sustainable development, zero deforestation, and environmental recuperation should not be too high, nor is there much reason to be euphoric about the possibilities of the conservation impact at the scale of innovations and new technologies.

The recognition of the somewhat disappointing long-term data and trends invites for a more realistic view of the promises of well-intended policies and economic initiatives to preserve the social and ecological diversity of the Amazon region while unlocking the region’s economic potential. Untangling growth and social and environmental decline require much stronger, more coherent, and continuous actions across all sectors of society, which necessitates a change of people’s behavior to pursue individual interests quickly. It requires systemic approaches that are aimed at changing the mindsets and aspirations not only of economic and political leaders but of millions of people in all parts of the world, especially in wealthy countries. This is perhaps the most difficult challenge, as it requires adopting new and more drastic ways of thinking.

However, the evidence found of the impact of policies to conserve forests, and improve the livelihoods of thousands of poor rural families offers hope. Even more considering is that periods of strong expansion of cultivated lands at the expense of forests have always been followed by less forest-destructive periods, as this suggests that the capacity of the economic actors active in the region is latent but limited. For many companies and investors, the region’s infrastructural and environmental limitations make the Amazon less attractive than other, better-developed regions around the globe. To have an impact, therefore, it is sufficient to effectively control a limited number of economic actors, albeit powerful ones, and counter their lobbyists. Agro-industrial land uses, cattle ranching, hydro power, and mineral extraction should prioritize regions where the economic needs of businesses and consumers can be met more sustainably and with less harm. The Amazon region, on the other hand, needs its alternative development agenda to be designed from the perspective of and in collaboration with the natural resource users who live in the region and based on already-existing instruments such as the designation of protected areas, the recognition of indigenous rights, land reforms, market-based conservation incentives, as well as compensations to local people for protecting nature. The marginalization of social and environmental policies must be overcome in favor of consistent action in and across all other policy areas. Ultimately, it is necessary to give up the idea that the use of the Amazon’s land and resources can satisfy the consumption and profit expectations of those living in cities and industrial countries without further destroying the region’s cultural and environmental wealth. This realization is essential to making real progress in the urgent search for solutions to deal with climate change and reverse biodiversity loss, while protecting local values and cultural diversity.

## Footnotes

1 https://www.ibge.gov.br
